# Amino acid metabolism regulated by lncRNAs: the propellant behind cancer metabolic reprogramming

**DOI:** 10.1186/s12964-023-01116-1

**Published:** 2023-05-01

**Authors:** Qifan Hu, Yutong Li, Dan Li, Yi Yuan, Keru Wang, Lu Yao, Zhujun Cheng, Tianyu Han

**Affiliations:** 1 Jiangxi Institute of Respiratory Disease, The First Affiliated Hospital of Nanchang University, Nanchang City, 330006 Jiangxi China; 2 Jiangxi Clinical Research Center for Respiratory Diseases, Nanchang City, 330006 Jiangxi China; 3 China-Japan Friendship Jiangxi Hospital, National Regional Center for Respiratory Medicine, Nanchang City, 330200 Jiangxi China; 4 School of Basic Medical Sciences, Nanchang University, Nanchang City, 330031 Jiangxi China; 5 Nanchang Vocational University, Nanchang City, 330500 Jiangxi China; 6 Department of Critical Care Medicine, Medical Center of Anesthesiology and Pain, The First Affiliated Hospital of Nanchang University, Nanchang City, 330006 Jiangxi China; 7 School of Huankui Academy, Nanchang University, Nanchang City, 330031 Jiangxi China; 8 Department of Burn, The First Affiliated Hospital of Nanchang University, Nanchang City, 330006 Jiangxi China

**Keywords:** Long non-coding RNA, Cancer, Metabolic reprogramming, Amino acid metabolism

## Abstract

Metabolic reprogramming is one of the main characteristics of cancer cells and plays pivotal role in the proliferation and survival of cancer cells. Amino acid is one of the key nutrients for cancer cells and many studies have focused on the regulation of amino acid metabolism, including the genetic alteration, epigenetic modification, transcription, translation and post-translational modification of key enzymes in amino acid metabolism. Long non-coding RNAs (lncRNAs) are composed of a heterogeneous group of RNAs with transcripts of more than 200 nucleotides in length. LncRNAs can bind to biological molecules such as DNA, RNA and protein, regulating the transcription, translation and post-translational modification of target genes. Now, the functions of lncRNAs in cancer metabolism have aroused great research interest and significant progress has been made. This review focuses on how lncRNAs participate in the reprogramming of amino acid metabolism in cancer cells, especially glutamine, serine, arginine, aspartate, cysteine metabolism. This will help us to better understand the regulatory mechanism of cancer metabolic reprogramming and provide new ideas for the development of anti-cancer drugs.

Video Abstract

**Supplementary Information:**

The online version contains supplementary material available at 10.1186/s12964-023-01116-1.

## Background

**Fig. 1:**
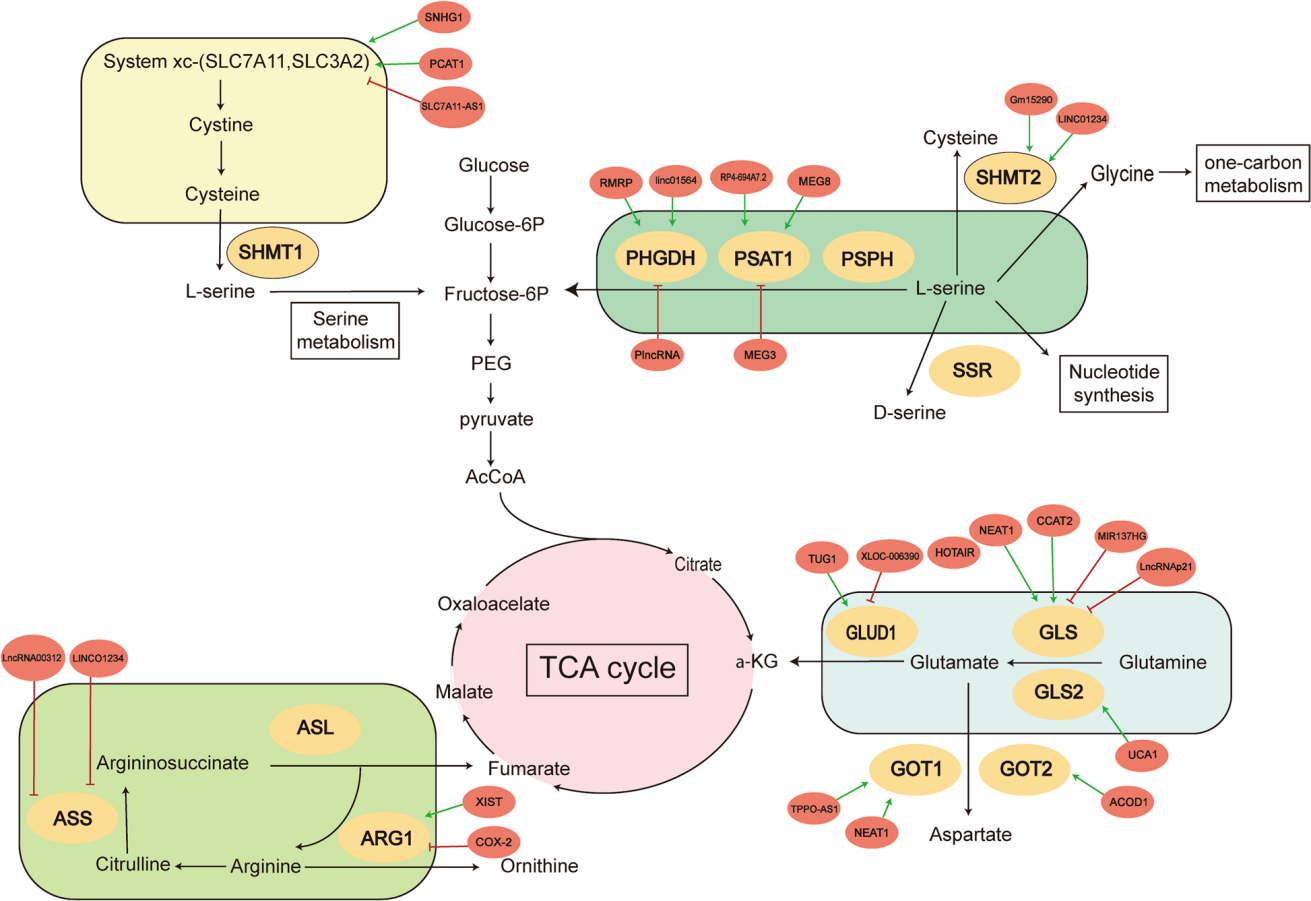
LncRNAs involved in the regulation of amino acid metabolism. LncRNAs can reprogram major amino acid metabolism pathways in cancer cells

Metabolic reprogramming is one of the main characteristics of cancer cells, which provide substrates and energy for the survival and proliferation of cancer cells [1, 2]. Aerobic glycolysis is one of the most representative metabolic modes that the majority of glucose was metabolized through glycolytic pathway to form lactate rather than oxidative phosphorylation even in the aerobic environment, a phenomenon also known as the Warburg effect [3]. The dysregulation of amino acids metabolism is another important aspect of cancer metabolism. Amino acid metabolism plays an important role in energy generation, nucleoside synthesis and maintaining redox homeostasis in cancer cells (Fig. 1) [4, 5]. For example, cancer cells utilize a large amount of glutamine to form α-ketoglutarate (α-KG) to replenish TCA cycle, which is depleted by aerobic glycolysis. Because the large demand of glutamine in cancer cells, glutamine is also called “conditionally essential amino acid”. In addition, other amino acids that provide carbon and nitrogen sources, such as serine, are also essential for cancer cells survival [6]. Elucidating the mechanism of cancer metabolic reprogramming is of great significance for understanding the mechanism of tumor formation and developing anti-tumor drugs.

**Fig. 2:**
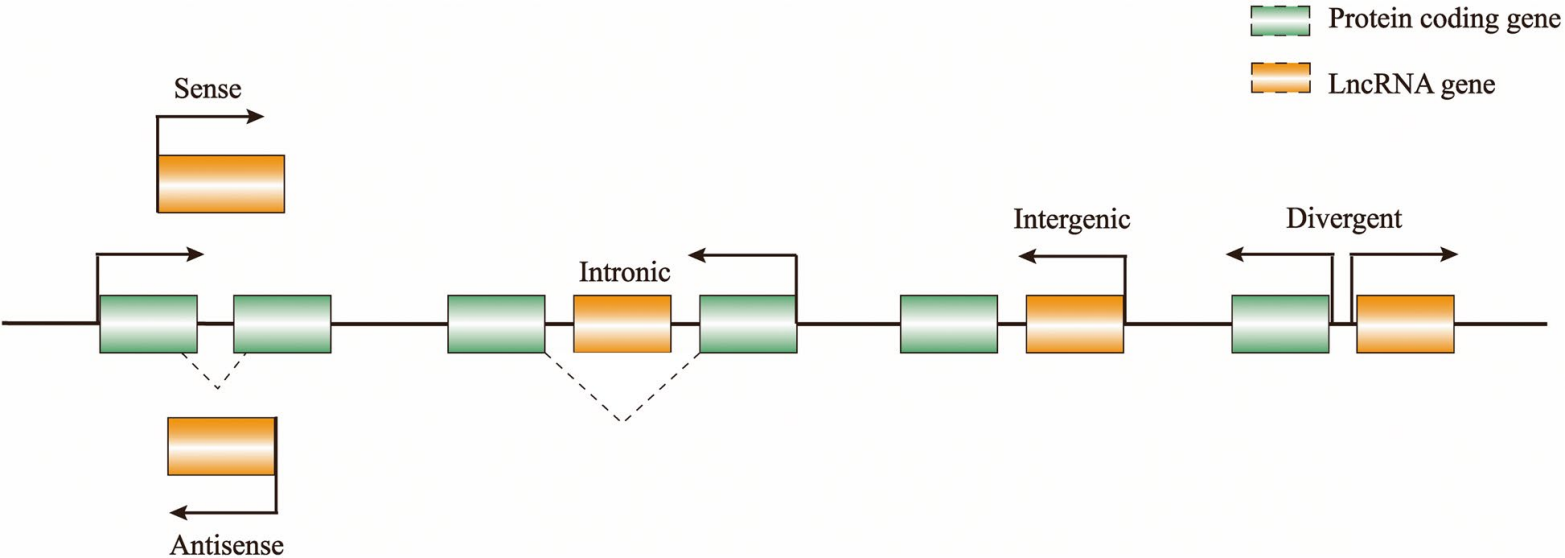
Classification of lncRNAs. According to the proximity of lncRNAs to protein-coding genes, they can be divided into the above five types

Long non-coding RNAs (lncRNAs) refers to a heterogeneous group of RNAs with transcripts of more than 200 nucleotides in length [7, 8]. LncRNAs make up a large portion of the transcriptome but can’t be translated into proteins or only into small peptides. They are generally transcribed by RNA polymerase II and undergo post-transcriptional RNA processing including 5’ capping, splicing and polyadenylation [9]. LncRNAs are localized both in the cytoplasm and nucleus, and their function usually depends on subcellular localization [10]. In addition, the location of lncRNAs can be changed when environmental transitions or infections [11]. There are five types of lncRNAs: (1) intronic lncRNAs; (2) intergenic lncRNAs; (3) antisense lncRNAs; (4) sense lncRNAs (5) divergent lncRNAs (Fig. 2) [12].

**Fig. 3:**
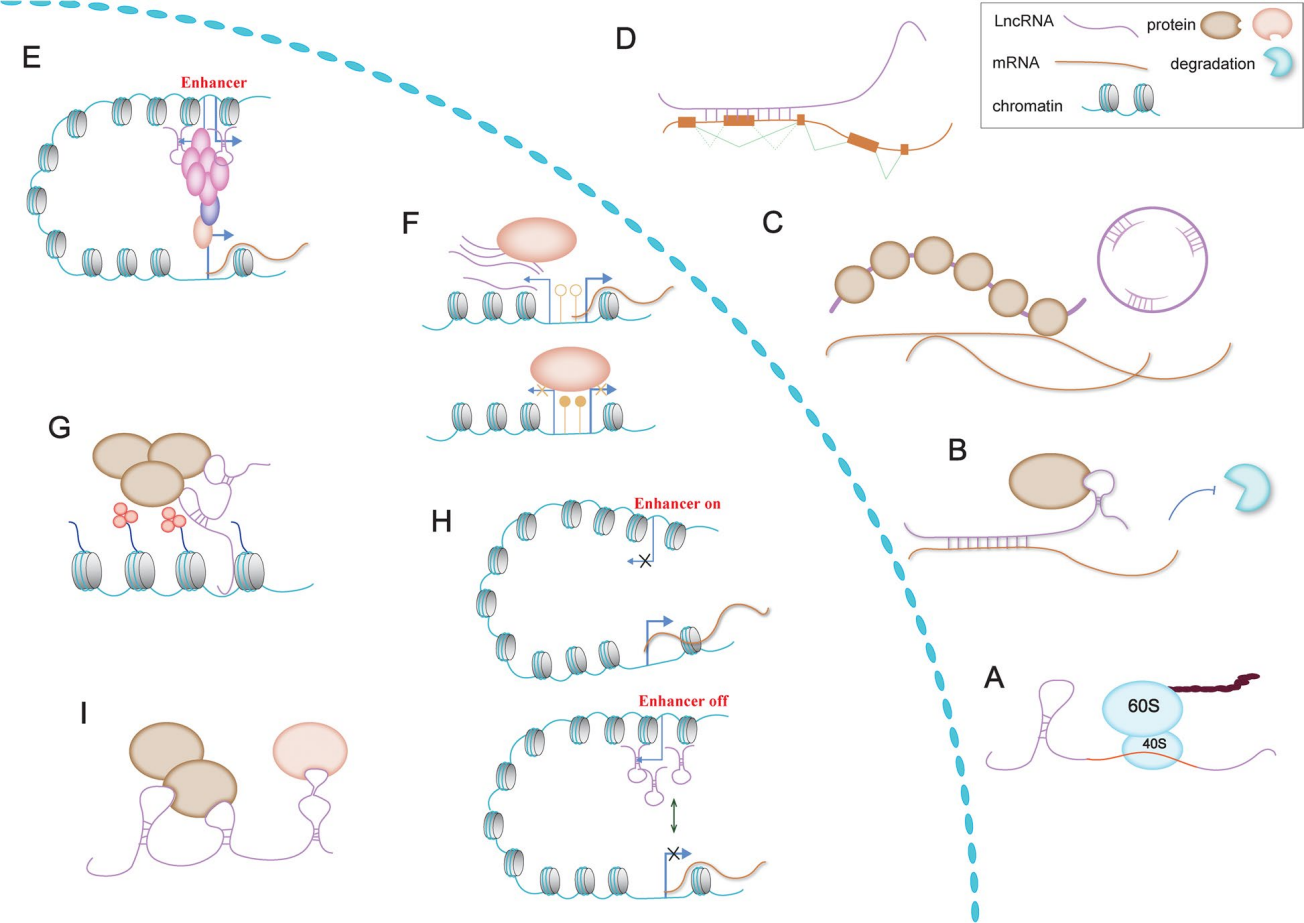
Mechanisms of lncRNAs function. **A** Some lncRNAs have short ORF regions. **B** LncRNAs can prevent mRNA degradation by recruiting specific proteins. **C** LncRNAs binding proteins to prevent or attenuate their binding to mRNAs. **D** LncRNAs binding to a primary RNA transcript and change the splicing pattern. **E** LncRNAs recruiting the mediator complex to an enhancer region for promote loop formation and transcription of target genes. **F** LncRNAs transcripts evicting proteins from chromatin to maintain a DNA-free methylation site for mRNA transcription. **G** LncRNAs can recruit specific proteins to target sites in the genome. **H** Some LncRNAs transcribed from an enhancer region can inhibit gene transcription process by interfering with enhancer contacting with promoter. **I** LncRNAs act as “scaffoldings” in the process of forming protein complexes

LncRNAs have been found but ignored in the last century. However, with the rapid development of whole genome sequencing technology and high-throughput sequencing technology, many lncRNAs have been found and their functions have been elucidated [13]. LncRNAs can bind to DNA, RNA, proteins [14–16] and regulate a broad spectrum of biological processes, including cell cycle, gene regulation, immune response, cell differentiation, post-transcriptional modification, and tumor metabolism [17–21]. LncRNAs regulate the function of target genes through the following four aspects (Fig. 3) [22]. (1) LncRNAs can act as “scaffoldings” in the process of protein complexes formation, facilitating the interplay between different signaling pathways, or bind to proteins to regulate their function and stability at the post-translational level [14]. (2) LncRNAs can bind to the promoter region of a gene to form DNA-RNA three-stranded heterozygous fragments, preventing the binding of transcription factors and the initiation of transcription process. LncRNAs can also act as “connecting tool” in the process of recruiting protein complexes with transcriptional regulation function to the promoter region of DNA, and performing functions such as chromatin reconstruction, activation or inhibition of transcriptional initiation, and epigenetic regulation [15]. (3) LncRNAs regulate the shearing, splicing, intracellular distribution and stability of mRNAs by direct binding. However, the binding between lncRNAs and miRNAs is usually more like a “sponge”, which reduces the binding between miRNA and target gene, thus reducing the inhibitory effect of miRNAs on target gene [16]. (4) Some lncRNAs have short ORF regions, which can be translated into short peptides to play their functions [23].

In recent years, the functions of lncRNAs in cancer cells has become a hot research area. Many researches have shown that lncRNAs can precisely affect the proliferation, differentiation, invasion and metastasis in cancer cells by regulating oncogenes or tumor suppressor genes, and are considered as potential tumor therapeutic targets [5, 24]. LncRNAs can also be used as clinical biomarkers for cancer diagnostics and prediction. Recently studies demonstrated that lncRNAs played an important role in regulating the transcription and translation of metabolism-related genes by acting as decoy molecules, scaffold molecules and competitive endogenous RNAs (ceRNAs), ultimately leading to metabolic reprogramming in cancer cells [25–27]. In this review, we focus on the important roles and function of lncRNAs in amino acid metabolism of cancer cells. We believe that a better understanding of lncRNA in amino acid metabolism will have breakthrough in cancer diagnosis and treatment.

## LncRNA and glutamine metabolism

Glutamine is the most abundant non-essential amino acid in human body, mainly concentrated in liver, kidney, skeletal muscle and brain tissue, and it is a precursor for the synthesis of many other amino acids, nucleotides and other important macromolecules [28]. In addition, glutamine can also activate mammalian target of rapamycin (mTOR) and maintain reactive oxygen species (ROS) homeostasis of cancer cells [29]. Glutamine is transported into cancer cells by the glutamine transporter, such as SLC7A8 and SLC7A5, or synthesize de novo in cancer cells. Then, glutamine was converted into glutamate and ammonium through glutaminase (GLS). Glutamate can be further converted into α-KG through glutamate dehydrogenase 1 (GLUD1), thus entering the TCA cycle to generate energy and intermediates for the synthesis of biological macromolecules [30]. Meanwhile, glutamate also acts as a substrate for metabolic enzymes to synthesis glutathione, which is a cellular antioxidant to help stabilize normal immune system function [31, 32]. The lack of glutamine will lead to endoplasmic reticulum stress response and protein misfolding, which induces cancer cells death [33].

Glutaminase is the initiating and rate-limiting enzyme of glutamine catabolism. Two genes encode glutaminase in the human genome. The *GLS* gene located on chromosome 2 encodes kidney-type glutaminase, which is mainly distributed in kidney, brain, pancreas and muscle tissue; *GLS2* gene located on chromosome 12 encodes liver-type glutaminase, mainly distributed in liver tissue [34]. The kidney-type glutaminase has two isoforms, exons 1–14 and 16–19 encode a longer form named KGA, and exons 1–15 encode a short form GAC, which has been demonstrated to be the main isoform in cancer cells [35, 36].

Several studies have shown that lncRNAs played an important role in glutamine metabolism. There are five lncRNAs bind to miRNA like a “sponge”, which reduces the binding between miRNA and GLS, thus reducing the inhibitory effect of miRNAs on GLS. Three of the lncRNAs include HOX transcript antisense intergenic RNA (HOTAIR), OIP5-AS1 and nuclear paraspeckle assembly transcript 1 (NEAT1) are highly expressed in different cancer cells compared to adjacent normal tissues [37–39]. These lncRNAs have similar regulatory mechanisms to upregulate GLS transcriptional levels, thus promoting the proliferation and migration of cancers cells. For example, lncRNA OIP5-AS1 can upregulate GLS mRNA expression level in melanoma cells by competitively sponge with GLS-binding miR-217 and reduced the inhibitory effect of miR-217-on GLS. These lncRNAs have potential as biomarkers for tumor diagnosis. LncRNAs can affect GLS expression not only in cancer cells but also in skin fibroblasts by competitively sponge microRNA. Researchers found that M2 macrophage can release lncRNA-ASLNC5088 into fibroblasts via exosomes. Then, ASLNC5088 can upregulate GLS mRNA expression by competitively sponge with GLS-binding miR-200c-3p at least 3 sites in skin fibroblasts. GW4869 as an exosome secretion inhibitor can reduce ASLNC5088 secretion to skin fibroblasts. Therefore, GW4869 can inhibit fibroblast over-activation and scar formation through regulate lncRNA-ASLNC5088/miR-200c-3p/GLS pathway [40]. From these studies, we concluded that lncRNAs regulated the expression of GLS by acting as ceRNAs competitively sponging GLS-binding miRNA in a variety of cancer cells and normal cells.

In addition to act as ceRNAs, lncRNAs can also regulate GLS expression in other ways. LncRNA HOTTIP is an oncogene and upregulate GLS expression in hepatocellular carcinoma (HCC). Meanwhile, researchers also found that miR-192 and miR-204 interfering with lncRNA HOTTIP expression via the Argonaute 2 (AGO2)-mediated RNA interference (RNAi) pathway can significantly suppresses GLS expression in HCC cell lines. In addition, Mir-192 /-204-HOTTIP-GLS axis regulate HCC cells proliferation and tumor formation. Therefore, lncRNA HOTTIP played a critical role in glutamine metabolism and HCC cells growth [41]. LncRNAs can also regulate GLS through the allele-specific manner. LncRNA colon cancer associated transcript 2 (CCAT2) regulates GLS mRNA through binding to the Cleavage Factor I (CFIm) complex with distinct affinities for the two subunits (CFIm25 and CFIm68). Allele-specific interactions between CCAT2, CFIm, and GLS pre-mRNA appear to result in the selection of the Poly (A) site within the GLS intron 14, leading to preferential splicing to the GAC isoform, which has higher catalytic activity. Therefore, CCAT2 can upregulate the expression of GAC and increase glutamine metabolism to promote the proliferation and migration of colon cancer cells [42]. In addition, lncRNAs can regulate glutamine metabolism by encoding small non-coding microRNAs. Heat Shock Factor 1 (HSF1) is an oncogene in colorectal cancer, which promotes mTOR activation and glutamine metabolism. HSF1 combine with DNA methyltransferase DNMT3a and recruits it to lncRNA MIR137 host gene (MIR137HG) promoter to inhibit the production of primary MIR137. Meanwhile, MIR137 can inhibit GLS protein expression. Therefore, HSF1 regulated DNMT3a/MIR137HG/MIR137 / GLS axis to raise glutaminolysis and mTOR activation and promote the process of colorectal cancer, and it is a potential therapeutic target for colorectal cancer [43]. LncRNAs can regulate GLS expression at transcriptional level too. Nuclear-enriched antisense lncRNA of glutaminase (GLS-AS) binds to GLS pre-mRNA via ADAR/Dicer-dependent RNA interference to form double-stranded RNA and inhibit GLS expression in pancreatic cancer cell lines. Overexpression of GLS-AS suppress the invasion and proliferation of pancreatic cancer cell lines by repressing the Myc/GLS pathway [44]. GLS2 can also be regulated by lncRNAs. LncRNA urothelial carcinoma-associated 1(UCA1) can function as a ceRNA to sequester miR-16 via binding it. As miR-16 binds to GLS and inhibits its transcription, UCA1 promoted the proliferation and migration of bladder cancer cells through acting as a ceRNA to upregulate GLS2 expression. In addition, overexpression of UCA1 could reduce intracellular ROS levels and protect bladder cancer cells from oxidative toxicity [45].

GLUD1 is another important enzyme in glutaminolysis, which converts glutamate to α-KG. GLUD1 was demonstrated to promote the proliferation and maintain redox homeostasis of cancer cells, and it is also a potential target for lncRNAs regulation. Researchers found that lncRNA taurine upregulated gene 1 (TUG1) downregulated miR-145 expression by acting as a ceRNA. MiR-145 bind to SIRT3 mRNA to suppress its expression, then SIRT3 expression reduce leads to GLUD1 downregulation through regulates GLUD1 acetylation level. TUG1 antagonizes Mir-145 and regulates Sirt3/GLUD1 axis, thereby affecting intrahepatic cholangiocarcinoma proliferation [46]. LncRNAs can also affect GLUD1 function via regulating key transcription factors. c-Myc could regulate the transcription of GLUD1 by targeting the promoter of GLUD1. The 5’UTR (1–772) and 3’UTR (3951–4899) of lncRNA XLOC_006390 bind to c-Myc and regulated it ubiquitination level, thereby affecting proteasome pathway degradation. Silencing lncRNA XLOC_006390 inhibited pancreatic cancer (PC) proliferation and migration by downregulating cellular α-KG levels [47].

## LncRNA and serine metabolism

Serine is another non-essential amino acid which is involved in nucleotide synthesis, oxidative stress response, TCA cycle and other metabolic processes. Serine can be obtained through extracellular import or the serine synthesis pathway, which is a branch pathway of glycolysis. First, phosphoglycolate dehydrogenase (PHGDH) catalyze glycolysis intermediate metabolite 3-phosphoglycolate (3-PG) into 3-phosphate hydroxypyruvate. Then, 3-phosphate hydroxypyruvate is catalyzed via phosphoserine aminotransferase (PSAT1) to produce 3-phosphoserine, followed by dephosphorylation via 1–3-phosphoserine phosphatase (PSPH) to produce serine. Serine can be further converted to glycine and 5,10-methylenetetrahydrofolate in cytoplasm or mitochondria by serine hydroxymethyl transferase (SHMT). Methyltetrahydrofolate dehydrogenase (MTFHD) can convert me-THF into 10-methylenetetrahydrofolate which is one of the sources in one-carbon metabolism. Recent studies showed that serine metabolism and related metabolic enzymes are indispensable in tumor initiation and progression. With the rapid development of whole genome sequencing technology and high-throughput sequencing technology, many lncRNAs was demonstrated to participate in serine metabolism.

PHGDH is the first rate-limiting enzyme for the serine biosynthetic pathway. Researchers found that PHGDH was upregulated in several cancers [48–50]. It has been reported that several lncRNAs functioned in cancer progression by targeting PHGDH. DDX3X (DEAD-Box Helicase 3 X-Linked) belongs to the Asp-Glu-Ala-Asp (DEAD) box protein family and has splicing and translation initiation functions, it is PHGDH mRNA-binding proteins. LncRNA RMRP (RNA Component of Mitochondrial RNA Processing Endoribonuclease) was shown to facilitate the recruitment of RNA binding DDX3X to 3’UTR of PHGDH mRNA. Meanwhile, overexpression of LncRNA RMRP or DDX3X can promote cisplatin resistance and spheroid formation in platinum-resistant ovarian cancer by increasing the translation of PHGDH mRNA. Therefore, the regulation of serine metabolism by RMRP / DDX3X /PHGDH signaling pathway is a potential therapeutic target for platinum-resistant ovarian cancer [51]. Another lncRNA PlncRNA-1 could inhibit the proliferation of breast cancer cells by promoting TNF-β protein expression and inhibiting PHGDH protein expression, suggesting that PHGDH functions as an oncogene in breast cancer [52]. Serine metabolism is closely related to glycolysis. Transcription factor ATF4 can interact with linc01564 and induce in response to glucose deprivation. Meanwhile, linc01564 can promote hepatocellular carcinoma cell proliferation (HCC) survival under glucose deprivation condition by regulating PHGDH expression in mRNA and protein levels. linc01564 as a ceRNA could interact with miR-107/miR-103a-3p at two binding sites. Then, miR-107/103a-3p can bind to PHGDH and inhibit its post-translational activity. Thereby, linc01564 facilitates serine metabolism and maintains ROS level under both glucose deprivation and normal conditions through the miR-107/103a-3p-PHGDH axis [53].

PSAT1 is another enzyme in serine synthesis pathway and reported to be regulated by lncRNAs. The expression of PSAT1 is inhibited by miR-15a-5p and miR-15b-5p in non-small cell lung cancer (NSCLC). The expression of lncRNA MEG8 is positively related to PSAT1 expression and serine synthesis, which acts as a ceRNA interacted with miR-15a-5p and miR-15b-5p [54]. LncRNA targeting PSAT1 can be found via GSE datasets (GSE94660 and GSE104310) which downloaded from the GEO. LncRNA RP4-694A7.2 can positively correlate with hepatocellular carcinoma proliferation and migration through binding to PSAT1 and promoting its expression [55]. LncRNAs also inhibited the expression of PSAT1. Epithelial-to-mesenchymal transition (EMT) is positively correlated with Gsk-3 β/snail signaling pathway, which promotes cancer progression. Overexpression of LncRNA maternally expressed gene 3 (MEG3) could downregulate PSAT1 and suppress the activation of GSK-3β/Snail signaling pathway in esophageal squamous cell carcinoma (ESCC). Therefore, MEG3 could inhibited ESCC proliferation by inhibiting PSAT1/ GSK-3 β/ Snail axis. In addition, lncRNA MEG3 could be used as a biomarker in ESCC diagnosis [56].

Serine and glycine are two nonessential amino acids which contribute to the main sources of one-carbon metabolism [57]. SHMT is a key serine/glycine conversion enzyme. SHMT can be encoded by two genes: SHMT1 and SHMT2. SHMT1 protein location in the cytoplasm and SHMT2 in the mitochondria. Interestingly, SHMT2, but not SHMT1 expression is significantly increased in several cancers [58]. LncRNAs can also regulate serine metabolism by affecting SHMT2 expression. In lung cancer, the targets of miR-615-5p includes IGF2, SHMT2 and AKT2. LncRNA Gm15290 can interact with miR-615-5p and inversely correlated with miR-615-5p levels in lung cancer. Overexpression of Gm15290 can promote the proliferation of lung cancer by upregulating SHMT2 expression [59]. Similarly, LncRNA LINC01234 is significantly upregulated in colon cancer. LINC01234 knockdown suppressed SHMT2 expression at mRNA and protein levels by acting as a ceRNA molecular sponge of miR-642a-5p, and serine/glycine metabolism could be inhibited via LINC01234 knockdown. In addition, researchers found that LINC01234 could act as a potential therapeutic target and biomarker for colon cancer [60].

A lack of serine in the diet may promote the conversion of glucose to serine, and cancer cells reprogram serine metabolism to satisfy their survival needs. Meanwhile, genes expression in the serine synthesis pathway are upregulated in a variety of cancers, such as increased gene copy number for PHGDH in melanoma and triple negative breast cancer [61]. LncRNAs plays an important role in the regulation of various enzymes in the serine synthesis pathway, and functions in tumor progression by regulating serine metabolism. Therefore, it is important to further study the regulation of lncRNAs on serine metabolism.

## LncRNA and arginine metabolism

Arginine is synthesized from citrulline through a two-step process, catalyzing by argininosuccinate synthase 1(ASS1) and argininosuccinate lyase (ASL). Then, arginase (ARG1) converts arginine to ornithine and urea. Ornithine is converted to citrulline for recycling in the mitochondria by ornithine transcarbamoylase (OTC). Although there are few studies on lncRNAs in regulating arginine metabolism, it may become a research hotspot in the future because of the importance of arginine metabolism in cancer.

ASS1 catalyzes the formation of argininosuccinate from aspartate, citrulline and ATP, it is responsible for the biosynthesis of arginine with ASL in most body tissues [62]. ASS1 is a rate-limiting enzyme in the arginine synthesis pathway, which is severely reduced or absent in some types of aggressive cancers, thus exhibiting exogenous arginine dependence [63]. Two studies have found that lncRNAs affected the expression of ASS1. In renal cell carcinoma (RCC), the expression level of lncRNA00312 was lower than adjacent normal tissues. Studies have shown that miR-34a-5p can bind and negatively regulate ASS1 expression [64]. LncRNA00312 inhibited RCC proliferation and invasion by promoting miR-34a-5p expression and inhibiting ASS1. ASS1 as a tumor suppressor gene may be a potential therapeutic target for RCC [65]. ASS1 is a key link between arginine metabolism and aspartate metabolism. In 2022, researchers found that lncRNA LINC01234 could promote HCC proliferation, migration and invasion by regulating aspartate metabolic reprogramming. Overexpression of LINC01234 could directly form an RNA–DNA complex with the ASS1 promoter, reduce the enrichment of transcription factor p53 on ASS1, and inhibit the expression of ASS1 at the protein and mRNA levels. Increased expression of LINC01234 led to the accumulation of celluar aspartate levels in HCC and increased mTOR activity [66].

Arginine is a non-essential amino acid for normal tissues, but many malignant tumor cells (such as melanoma, liver cancer, etc.) have not expression ASS1 and cannot synthesize arginine, so arginine is an essential amino acid for the above malignant tumor cells [67, 68]. Arginase can catalyze the hydrolysis of arginine, so arginase does not affect the growth of normal cells but inhibits the growth of tumor cells [69]. At present, amino acid deprivation has become a new method to therapy cancer, and the different tolerance to arginine deprivation between tumor cells and normal cells can be used to specifically inhibit the growth of cancer cells. LncRNAs can affect tumor associated macrophages (TAM) by regulating Arg1 expression. Arg1 is a specific marker for M2 macrophage. Several oncogenic lncRNAs can promote tumor progression by affecting M2 polarization and Arg1 expression, such as lncRNA AK0363962, lncRNA CRNED, lncRNA X inactive specific transcript (XIST), lncRNA NEAT1, lncRNA runt-related transcription factor 1 overlapping RNA (RUNXOR) [70–74]. For example, overexpression of lncRNA XIST downregulated the expression of Arg1 and promote M2 macrophages polarization. Conversely, several tumor suppressor lncRNAs could inhibited cancer cells survival, such as lncRNA cox-2 [75]. LncRNA cox-2 inhibits immune evasion, proliferation and migration in HCC by inhibiting Arg1 and the polarization of M2 macrophages. These lncRNAs have the potential to improve tumor immunotherapy.

## LncRNA and aspartate metabolism

As one of the lowest concentrations of amino acids in the blood, aspartate is involved in the synthesis of proteins and nucleotides and therefore plays an important role in cells growth [76]. Aspartate has low circulating levels compared to other amino acids, and cancer cells reprogram aspartate metabolism to support cell growth [77]. Under the condition of enough oxygen, aspartate is synthesized from oxaloacetic acid (OAA) and L-glutamic under the action of glutamate oxaloacetate transaminase 1 (GOT1). However, aspartate synthesis is blocked under hypoxic tumor microenvironment and aspartate is a key factor in tumor proliferation [78].

The effects of lncRNAs on aspartate metabolism mainly focuses on the regulation of GOT expression. GOT is a key enzyme linked to aspartate metabolism and carbohydrate metabolism, and is mainly distributed in tissues such as heart, liver, skeletal muscle and kidney. This enzyme not only regulates amino acid metabolism, but also promotes cancer cells proliferation by maintaining redox balance. There are two genotypes of GOT in cells: GOT1 protein located in cytoplasm, and GOT2 in mitochondria. In addition, GOT1 also participates in ferroptosis mechanism. Many studies showed that lncRNA TPPO-AS1 acted as a tumor motivator in various cancers. TPPO-AS1 could act as a molecular sponge that bound to miR-429 and suppress its expression. Furthermore, miR-429 directly bind to GOT1 mRNA and negatively regulated its expression. By regulating Mir-429 / GOT1 axis, TPPO-AS1 promoted HCC progression [79]. LncRNA NEAT1 can regulate GOT1 and transferrin receptor (TFRC) expression during ferroptosis. Compared with normal Cells, more exosome-packaged NEAT1 crossed blood–brain barrier (BBB) into sepsis-induced ferroptosis cells. MiR-9-5p has binding sites with NEAT1, TFRC mRNA. Then, NEAT1 acted as a ceRNA by sponging miR-9-5p to upregulate the expression of TFRC and GOT1 [80].

As another enzyme in aspartate metabolism, GOT2 also involved in many processes except for aspartate metabolism, such as long-chain fatty acid uptake and TCA cycle [81, 82]. The regulation of GOT2 by LncRNAs in cancer cells has not been reported so far. However, researchers found that virus-induced lncRNA mediated metabolic could affect GOT2 function. Researchers found that knockdown lncRNA-ACOD1 significantly reduced vesicular stomatitis virus (VSV), vaccinia virus (VACV) and herpes simplex virus type 1 (HSV-1) infection in macrophages. Overexpression of GOT2 promoted viral infection in macrophages by upregulating lncrNA-ACOD1. Meanwhile, lncRNA-ACOD1 interacted GOT2 with at 15-residue peptide (residues 54 to 68) and simulating GOT2 activity and its metabolites. This is a novel feedback way during viral infection and lncRNA-ACOD1-GOT2 interaction network was a potential therapeutic target for viral infection [83].

## LncRNA and cysteine metabolism

Cysteine is a non-essential amino acid, which provides carbon source for TCA cycle, participates in the synthesis of glutathione (GSH) to maintain redox balance, and generates hydrogen sulfide to promote the production of ATP through its sulfhydryl group [84]. Thus, cysteine plays an important role in oxidative stress, energy metabolism, ferroptosis and autophagy. Cysteine in cancer cells is mainly derived from the transport of cystine transporters (consist of light chain SLC7A11 and heavy chain SLC3A2) and the synthesis of endogenous sulfur transfer pathways. Studying the mechanism of cysteine metabolism during cancer process provides the possibility for developing cancer diagnostic tools and targeted drugs. Therefore, it is of great significance to study the effect of lncRNAs regulating cysteine metabolism on cancer cells.

SLC7A11 is known to inhibit ferroptosis by promoting GSH synthesis through the uptake of cysteine. Recent studies found that SLC7A11 is highly expressed in non-small cell lung cancer, oral cancer, prostate cancer, malignant glioma and other cancers, which is closely related to cancer proliferation, invasion, metastasis and drug resistance [85]. The regulation of SLC7A11 by lncRNAs achieved many important results. LncRNA SLC7A11-AS1 regulates the expression of SLC7A11 at mRNA and protein levels in a variety of tumors. SLC7A11-AS1 could be a potential therapeutic target in a variety of tumors. In 2017, Xiao et al. first reported the effect of SLC7A11-AS1 on tumor progression. The researchers found that SLC7A11-AS1 expression was significantly reduced in gastric cancer tissues compared with adjacent nontumor tissues. And SLC7A11-AS1 can as a biomarker in the diagnosis of gastric cancer (GC). Knockdown of SLC7A11-AS1 can promote the expression of SLC7A11 at transcriptional level through the ASK1-P38MAPK /JNK signaling pathway, thus inhibiting the proliferation of GC cells [86]. SLC7A11-AS1 also involved in drug resistance in GC. SLC7A11 is play an important role in intracellular redox balance and GSH synthesis. Accumulating evidence suggests that SLC7A11 is upregulated can lead to multidrug resistance in colorectal cancer. Increased expression of miR-33a-5p can reduce SLC7A11 by interacting with SLC7A11 at 3’UTR sequences. SLC7A11-AS1 regulates the expression of SLC7A11 by binding to miR-33a-5p. Silencing SLC7A11-AS1 can reduce intracellular ROS level and increase intracellular GSH level by regulating miR-33a-5p / SLC7A11 axis thus, SLC7A11-AS1 have function in cisplatin resistance in GC [87]. Meanwhile, SLC7A11-AS1 knockdown promoted ovarian cancer proliferation by upregulating SLC7A11 [88]. In addition, overexpression of SLC7A11-AS1 could promote TGF-β-mediated HCC invasion and metastasis [89]. Conversely, SLC7A11-AS1 is highly expressed in pancreatic ductal adenocarcinoma (PDAC) compared with adjacent nontumor tissues. In gemcitabine-resistant PDAC cells, the intracellular level of ROS is reduced via overexpression of SLC7A11-AS1. SLC7A11-AS1 Exon 3 (440–1,725 nt of SLC7A11-AS1) binds to the E3 ubiquitin ligase SCF^β −TRCP1^ and blocked its function, thus SCFβ ^β−TRCP1^ inhibits ubiquitination and proteasomal degradation of NRF2. Therefore, SLC7A11-AS1 promotes PDAC stemness and chemoresistance through SCF^β −TRCP1^/NFR2 signaling axis regulation of intracelluar ROS levels [90]. In adition, SLC7A11-AS1 as a ceRNA promoted the expression of TRAIP by binding to miR-4775 and inhibits its expression in lung cancer cells. Overexpression of SLC7A11-AS1 promoted lung cancer proliferation, migration and invasion [91]. Therefore, the regulatory mechanism of SLC7A11-AS1 are different in several cancers.

In addition to SLC7A11-AS1, there are several other lncRNAs can regulate SLC7A11 and affect cysteine metabolism in cancer cells. In prostate cancer, researchers found that lncRNA SNHG3, lncRNA prostate cancer-associated transcript 1 (PCAT1) and lncRNA OIP5-AS1 could affect SLC7A11 mRNA expression through different regulatory mechanisms. The three lncRNAs were significantly overexpressed in PC tissues compared with adjacent normal tissues, and promoted PC proliferation, migration and inhibit PC apoptosis, ferroptosis by promoting SLC7A11 expression. MiR-128 can bind to SLC7A11 and suppress its mRNA expression. OIP5-AS1 acted as a ceRNA to upregulate the expression of SLC7A11 at the posttranscriptional level via competitively binding to miR-128–3, thus inhibiting Cd-induced ferroptosis in PC cells. In addition, OIP5-AS1 has the potential to be a biomarker for PC diagnosis. Similarly, overexpression of SNHG3 promotes SLC7A11 through acted as a sponge of miR-152-3p. MiR-152-3p and miR-128–3 can bind to SLC7A11 and constrained its expression in PC cells. Knockdown of transcription factor TFAP2C inhibited PCAT1 mRNA levels in PC cells. Meanwhile, PCAT1 affected SLC7A11 through two regulatory mechanisms. On the one hand, PCAT1 (1093–1367 nt) bound to c-Myc (151–202 amino acid) and inhibited its proteasome degradation, thus increasing SLC7A11 mRNA expression is upregulated by promoting c-Myc expression. On the other hand, PCAT1 functioned as a ceRNA to regulate SLC7A11 mRNA expression by sponging miR-25-3p. MiR-25-3p constrained SLC7A11 expression in PC cells. These lncRNAs might play an important role in PC therapy [92–94]. LncRNA LINC00618 promoted leukemia apoptosis and ferroptosis by regulating SLC7A11 at posttranscriptional level and increases the expression of BAX and cleaved caspase 3. Meanwhile, LNC00618 could bind to lymphoid-specific helicase (LSH) and inhibited it to regulate SLC7A11 transcription [95]. LncRNAs often acted as a sponge of miRNA to promote SLC7A11 expression at mRNA and protein levels, such as lncRNA UC.339 / miR-339 /SLC7A11 axis promoted lung adenocarcinoma proliferation, migration and invasion by inhibiting ferroptosis [96]. Similar mechanisms were also observed, for lncRNA ADAMTS9-AS1 / miR-5887 /SLC7A11 axis in epithelial ovarian cancer [97], and lncRNA SLC16A1-AS1 / miR-143-3p/SLC7A11 axis in renal cancer [98].

SLC3A2 is responsible for maintaining the stability and membrane positioning of the cysteine transporters. In addition, SLC3A2 can also form a polymer with other amino acid transporters, such as an amino acid-polyamine-organic cation transporter jointly formed with SLC7A5, which can catalyze the transmembrane transport of thyroid hormones, drugs and hormone precursors [99]. The effect of lncRNAs on SLC3A2 plays an important role in regulating cysteine metabolism. LncRNA small nucleolar RNA host gene 1 (SNHG1) was significantly overexpressed in several tumor tissues compared with adjacent normal tissues, and it promoted the proliferation of a variety of tumors, such as lung cancer, colorectal cancer and hepatocellular carcinoma. The regulatory mechanism was that SNHG1 can promote SLC3A2 transcription by directly binding the Mediator complex to facilitate enhancer-promoter. ALC3A2 could contribute to adhesion-induced signaling and activate to FAK and phosphoinositide-3 kinase (PI3K). Thus, SNHG1 can phosphorylation PI3K downstream target AKT and activate AKT signaling pathway by promote SLC3A2 mRNA transcription in H1299 and HCT116 cell lines. In addition, FUBP1 interplays with FIR and TFIIH to affect MYC gene expression, SNHG1 could reduce the binding of FUBP1 to FIR, thus regulating the transcription of c-Myc [100]. Researchers found that the translocation of miR-21 to the nucleus induced by Sorafenib could promote SNHG1 expression by binding to SNHG1 and promoting its transcription in HCC cells [101] (Tables 1, 2, 3, 4).

**Table 1:** LncRNAs involving glutamine metabolism

LncRNAs	Expression	Genes and Pathways	Cancer	References
HOTAIR	↑	miR-126-5p, GLS	gliomas	[37]
OIP5-AS1	↑	miR-217, GLS	melanoma	[38]
NEAT1	↑	miR-23a-3P, GLS	medulloblastoma	[39]
ASLNC5088	↑	miR-200c-3p, GLS	skin fibroblasts	[40]
HOTTIP	↑	miR-192, miR-204, GLS	hepatocellular carcinoma	[41]
CCAT2	↑	CFlm, GLS	colon cancer	[42]
MIR137HG	↓	MIR137, GLS	colorectal cancer	[43]
GLS-AS	↓	ADAR, GLS	pancreatic cancer	[44]
UCA1	↑	miR-16, GLS2	bladder cancer	[45]
TUG1	↑	MiR-145, SIRT3, GLUD1	intrahepatic cholangiocarcinoma	[46]
XLOC_006390	↓	c-Myc, GLUD1	pancreatic cancer	[47]

**Table 2:** LncRNAs involving serine metabolism

LncRNAs	Expression	Genes and Pathways	Cancer	References
RMRP	↑	DDX3X, PHGDH	ovarian cancer	[51]
PlncRNA	↓	TNF-β, PHGDH	breast cancer	[52]
linc01564	↑	ATF4, miR-107, miR-103a-3p, PHGDH	hepatocellular carcinoma	[53]
MEG8	↑	miR-15a-5p-miR-15b-5p/PSAT1	Non-small cell lung cancer	[54]
RP4-694A7.2	↑	PSAT1	hepatocellular carcinoma	[55]
MEG3	↓	GSK-3β/Snail, PSAT1	esophageal squamous cell carcinoma	[56]
Gm15290	↑	miR-615-5p, SHMT2	lung cancer	[59]
LINC01234	↑	miR-642a-5p, SHMT2	colon cancer	[60]

**Table 3:** LncRNAs involving arginine metabolism

LncRNAs	Expression	Genes and Pathways	Cancer	References
lncRNA 00,312	↓	miR-34a-5p, ASS1	renal cell carcinoma	[64]
LINC01234	↓	ASS1, P53	hepatocellular carcinoma	[65]
AK036396	↑	Fcnb, Arg1	Lung cancer	[70]
CRNED	↑	Arg1	liver cancer	[71]
XIST	↑	Arg1	lung cancer	[72]
NEAT1	↑	Arg1	thyroid carcinoma	[73]
RUNXOR	↑	Arg1	lung cancer	[74]
cox-2	↓	Arg1	hepatocellular carcinoma	[75]

**Table 4:** LncRNAs involving cysteine metabolism

LncRNAs	Expression	Genes and Pathways	Cancer	References
SLC7A11-AS1	↓	ASK1-P38 MAPK, JNK, SLC7A11	gastric cancer	[86]
SLC7A11-AS1	↓	miR-33a-5p	gastric cancer	[87]
SLC7A11-AS1	↓	SLC7A11	ovarian cancer	[88]
SLC7A11-AS1	↓	TGF-β	gastric cancer	[89]
SLC7A11-AS1	↑	SCFβ -TRCP1/NFR2	pancreatic ductal adenocarcinoma	[90]
SLC7A11-AS1	↑	TRAIP	lung cancer	[91]
PCAT1	↑	miR-4475, SLC7A11 c-Myc	prostate cancer	[92]
SNHG3	↑	SLC7A11, miR-152-3p	prostate cancer	[93]
OIP5-AS1	↑	SLC7A11	prostate cancer	[94]
LINC00618	↓	LSH, SLC7A11	leukemia	[95]
UC.339	↑	miR-339, SLC7A11	lung adenocarcinoma	[96]
ADAMTS9-AS1	↑	miR-5887, SLC7A11	ovarian cancer	[97]
SLC16A1-AS1	↑	miR-143-3p, SLC7A11	renal cancer	[98]
SNHG1	↑	SLC3A2, AKT	lung cancer/colorectal cancer	[100]
SNHG1	↑	miR-21	hepatocellular carcinoma	[101]

## Conclusions

Cancer cells metabolic reprogramming has been studied for more than 100 years since Otto Warburg discovered the Warburg effect in 1920. It is one of the key features in tumorigenesis and tumor progression. Many studies have shown that in addition to abnormal glucose metabolism, amino acid metabolism is also an important research area of cancer cells metabolic reprogramming. Studies on the functions of metabolism-related lncRNAs can help us better understand the regulatory mechanism of cancer. The function of these lncRNAs plays a key role in tumorigenesis, progression and metabolic reprogramming. With the development of biotechnology, it has become easier to study the role of lncRNA-mediated metabolic reprogramming. In this review, we focused on the effect of lncRNA-mediated amino acid metabolism on tumor progression. We found that lncRNAs regulates amino acid metabolism mainly by affecting the expression of metabolism -related enzymes and amino acid transporters. LncRNAs are involved in regulation of gene expression at various levels, including epigenetic modification, transcriptional regulation, RNA splicing, nuclear shuttle, post-transcriptional level, translational level and post-translational level, basically running through all the currently known regulatory levels in the body. In addition, LncRNAs can act as endogenous competing RNA-competitive sponge miRNAs, thereby inhibiting the regulation of target genes by miRNAs.

Studies shown that lncRNAs have been used as biomarkers in a variety of cancers. However, the current research on lncRNAs is still in its infancy, and there are still many issues need to be discussed: (1) The secondary structure, function and molecular mechanism of lncRNA are not fully studied. (2) Current studies on the effect of lncRNAs on tumor metabolism are not comprehensive, especially the precise mechanisms of lncRNAs on key enzymes at multiple levels (transcription, translation and post-translational modification). (3) Current studies on lncRNAs are mostly at the experimental stage, and there is still a long way to go for clinical application. In conclusion, exploring lncRNAs-mediated regulation of amino acid metabolism will contribute to a deeper understanding of tumorigenesis and the vulnerability of tumor cells. This will provide theoretical basis for developing anti-cancer drugs and exploring potential biomarkers for cancer diagnosis.

## Data Availability

Not applicable.
